# Control of triple-negative breast cancer using ex vivo self-enriched, costimulated NKG2D CAR T cells

**DOI:** 10.1186/s13045-018-0635-z

**Published:** 2018-07-06

**Authors:** Yali Han, Wei Xie, De-Gang Song, Daniel J. Powell

**Affiliations:** 1 Ovarian Cancer Research Center, Department of Obstetrics and Gynecology, Perelman School of Medicine, University of Pennsylvania, 3400 Civic Center Blvd, Smilow CTR, Philadelphia, PA 19104 USA; 2 Department of Radiation Oncology, Qilu Hospital of Shandong University, Jinan, 250012 China; 3 Center for Stem Cell Research and Application, Union Hospital, Tongji Medical College, Huazhong University of Science and Technology, Wuhan, 430022 China; 4 Department of Pathology and Laboratory Medicine, Abramson Cancer Center, Perelman School of Medicine, University of Pennsylvania, 3400 Civic Center Blvd, Rm 8-103 Smilow CTR, Philadelphia, PA 19104 USA; 5 Present address: Janssen R&D, LLC, 1400 McKean Road, Spring House, PA 19477 USA

**Keywords:** Chimeric antigen receptor, T cells, NKG2D ligands, Immunotherapy, Triple-negative breast cancer

## Abstract

**Background:**

Triple-negative breast cancer (TNBC) is an aggressive disease that currently lacks effective targeted therapy. NKG2D ligands (NKG2DLs) are expressed on various tumor types and immunosuppressive cells within tumor microenvironments, providing suitable targets for cancer therapy.

**Methods:**

We applied a chimeric antigen receptor (CAR) approach for the targeting of NKG2DLs expressed on human TNBCs. Lentiviral vectors were used to express the extracellular domain of human NKG2D that binds various NKG2DLs, fused to signaling domains derived from T cell receptor CD3 zeta alone or with CD27 or 4-1BB (CD137) costimulatory domain.

**Results:**

Interleukin-2 (IL-2) promoted the expansion and self-enrichment of NKG2D-redirected CAR T cells in vitro. High CD25 expression on first-generation NKG2D CAR T cells was essential for the self-enrichment effect in the presence of IL-2, but not for CARs containing CD27 or 4-1BB domains. Importantly, self-enriched NKG2D CAR T cells effectively recognized and eliminated TNBC cell lines in vitro, and adoptive transfer of T cells expressing NKG2D CARs with CD27 or 4-1BB specifically enhanced NKG2D CAR surface expression, T cell persistence, and the regression of established MDA-MB-231 TNBC in vivo. NKG2D-z CAR T cells lacking costimulatory domains were less effective, highlighting the need for costimulatory signals.

**Conclusions:**

These results demonstrate that CD27 or 4-1BB costimulated, self-enriched NKG2D CAR-redirected T cells mediate anti-tumor activity against TNBC tumor, which represent a promising immunotherapeutic approach to TNBC treatment.

**Electronic supplementary material:**

The online version of this article (10.1186/s13045-018-0635-z) contains supplementary material, which is available to authorized users.

## Background

Triple-negative breast cancers (TNBC), an aggressive form of breast cancer that lacks significant expression of the human epidermal growth factor receptor 2 (HER2), estrogen receptor (ER), and progesterone receptor (PR), accounts for approximately 15~20% of invasive breast cancers. In the absence of obvious targets, patients with TNBC do not benefit from endocrine therapy or other available targeted agents [1]. To date, the standard treatment still depends on surgery and adjuvant chemotherapy and radiotherapy. Patients with TNBC have a worse outcome after chemotherapy, compared to breast cancers patients with other subtypes [2], a finding that reflects the intrinsically adverse prognosis associated with the disease. Thus, effective therapeutic strategies are urgently needed for TNBC patients.

Cancer cells including TNBC cells frequently upregulate “stress” induced ligands recognized by the NK cell activating receptors NKG2D (natural-killer group 2, member D) and DNAM-1(CD226) [3, 4]. Therefore, the adoptive transfer of NK cells may represent a promising treatment strategy for these cancers. Large numbers of autologous NK cells can be infused to patients but often do not mediate tumor regression [5]. The feasibility of targeting NKG2D ligands (NKG2DLs) utilizing chimeric antigen receptor (CAR) engineered T cell approach was demonstrated by Sentman and colleagues [6] in 2005, and early clinical trial results now show the significant promise of this approach [7]. This CAR construct contained the full-length NKG2D fused to the cytoplasmic domain of CD3z with costimulation provided endogenously by Dap10. We and others [8, 9] have utilized 4-1BB or CD28 signaling platform-based NKG2D CAR T cells, in which the NKG2D extracellular domain (ECD) was connected to a transmembrane portion of the platform in a reverse orientation that maintained the ligand binding specificity and function. In these CARs, the human NKG2D ECD recognize several distinct ligands, including the MIC (MHC class I-related chain) family and six members of the ULBP/RAET (UL16-binding protein, or retinoic acid early transcript) family [10], which are generally absent or expressed at low levels by healthy tissues but widely expressed on cancer cells. In the present study, we identify for the first time NKG2DL-expressing TNBCs as being sensitive to NKG2D CAR T cell attack, offering a new strategy for effective therapy. Further, we identify an unexpected role for IL-2 in the self-enrichment of first-generation NKG2D CAR T cells as well as the impact of 4-1BB and CD27 costimulatory signaling domains in NKG2D CARs in generating more potent T cells against TNBCs in vitro and in vivo.

## Methods

### Cell lines

Human cell lines used in immune-based assays include the established human breast cancer cell line MCF7 and TNBC cell lines MDA-MB-231, MDA-MB-436, MDA-MB-468, MDA-MB-453, and BT549. The mouse malignant mesothelioma cell line, AE17 (kindly provided by Steven Albelda, University of Pennsylvania), was used as antigen negative control. For bioluminescence assays, the cancer cell lines were transfected to express firefly luciferase (fluc). Lentivirus packaging was executed using the immortalized normal fetal renal 293T cell line purchased from ATCC. All cell lines were maintained in complete medium: RPMI-1640 supplemented with 10% heat inactivated FBS, 100 U/ml penicillin, and 100 mg/ml streptomycin sulfate.

### CAR construction and lentivirus production

The NKG2D CAR constructs are comprised of the extracellular portion of human NKG2D (aa 82–216) linked to a CD8a hinge and transmembrane region, followed by a CD3z signaling moiety alone (NKG2D-z) or in tandem with the 4-1BB or CD27 intracellular signaling motif, which was previously described [11, 12]. CAR sequences were preceded in frame by a green fluorescent protein (GFP) sequence followed by the 2A ribosomal skipping sequence.

High-titer replication-defective lentivirus were produced and concentrated as previously described [13]. Briefly, 293T cells were seeded in 150-cm^2^ flask and transfected using TurboFect (Life Technologies) according to the manufacturer’s instructions. NKG2D CAR transgene plasmid (15 μg) was co-transfected with 18 μg pRSV.REV (Rev expression plasmid), 18 μg pMDLg/p.RRE (Gag/Pol expression plasmid), and 7 μg pVSV-G (VSV glycoprotein expression plasmid) with 174-ul transfection reagent Express In (1 μg/ul) per flask. Supernatants were collected at 24 and 48 h after transfection, concentrated tenfold by ultracentrifugation for 2 h at 28,000 rpm with a Beckman SW32Ti rotor (Beckman Coulter). The viruses were aliquoted and stored at − 80 °C until ready to use for titering or experiments. All lentiviruses used in the experiments were from concentrated stocks.

### Human T cells and transfection

Primary human T cells, purchased from the Human Immunology Core at University of Pennsylvania, were isolated from healthy, normal donors following leukapheresis by negative selection. All T cell samples were collected under a protocol approved by a University Institutional Review Board, and written informed consent was obtained from each healthy, normal donor. T cells were cultured in R10 medium and stimulated with anti-CD3 and anti-CD28 monoclonal antibodies (mAb)-coated beads (Invitrogen). Approximately 18 to 24 h after activation, human T cells were transduced. Briefly, 0.5 × 10^6^ T cells were infected with a multiplicity of infection (MOI) 2 of the NKG2D receptor lentiviral vector and expanded for 2 weeks. Human recombinant interleukin-2 (IL-2; Novartis) was added every 2–3 days to a 50-IU/ml final concentration, and a cell density of 0.5 × 10^6^ to 1 × 10^6^ cells/ml was maintained. Engineered CAR T cells were rested in cytokine-free medium for 24 h and were then used for functional analysis.

### Flow cytometric analysis

The following fluorochrome-conjugated monoclonal antibodies, purchased from BD Biosciences, were used for T cell phenotypic analysis: APC-Cy7 anti-human CD3, FITC anti-human CD4, APC anti-human CD8, PE anti-human CD45, and APC anti-human NKG2D. PE anti-human CD137, APC anti-human PD-1, and Pacific Blue anti-human TIM-3 were purchased from Biolegend. 7-Aminoactinomycin D (7-AAD) was used for viability staining. Expression of NKG2D CAR was detected by GFP and surface NKG2D expression using anti-NKG2D Ab. For the in vivo experiments, peripheral blood was obtained via retro-orbital bleeding and stained for the presence of human CD45, CD4, and CD8 T cells. Gating specifically on the human CD45+ population, the CD4+ and CD8+ subsets were quantified using TruCount tubes (BD Biosciences) with known numbers of fluorescent beads as described in the manufacturer’s instructions.

NKG2DLs were analyzed using PE anti-MICA/B (clone 6D4, BD Pharmingen), PE anti-ULBP1 (clone 170818, R&D System), PE anti-ULBP2/5/6 (clone 165903, R&D System), anti-ULBP3 (clone 2F9, Santa Cruz), and polyclonal Per-CP-anti-human ULBP4 (R&D System). Recombinant human NKG2D Fc chimera and a control recombinant human folate receptor-alpha (FRA) Fc chimera were purchased from R&D System. Matched secondary and isotype antibodies were used in all analyses. Flow cytometry was performed on BD FACSCanto II flow cytometer, and flow cytometric data were analyzed using FlowJo Version 7.2.5 software.

### Cytokine release assays

Cytokine release assays were performed by co-culturing 1 × 10^5^ T cells with 1 × 10^5^ target cells in triplicate in a 96-well flat bottom plate in a total volume of 200-ul R10 media. After 20~24 h, cells free co-culture supernatants were collected and ELISA (Biolegend, San Diego) was performed, according to manufacturer’s instructions, to measure the secretion of IFN-γ. The values shown represent the mean of triplicate wells.

### Cytotoxicity assays

For cell-based bioluminescence assays, 5 × 10^4^ firefly luciferase (fLuc)-expressing tumor cells were cultured with R10 media in the presence of different T cell ratios in a 96-well microplate (BD Biosciences). After incubation for ~ 20 h at 37 °C, each well was filled with 50 ul of d-luciferin (0.015 g/ml) resuspended with PBS and imaged with the Xenogen IVIS Spectrum. Tumor cell viability percentage was calculated as the mean luminescence of the experimental sample minus background divided by the mean luminescence of the input number of target cells used in the assay minus background times 100. All data are represented as a mean of triplicate wells.

Additional cytotoxicity of NKG2D CAR-T cells was measured in real time using an xCELLigence (ACEA Bioscience) label-free, impedance-based cell sensing device. AE17, MDA-MB-436, MDA-MB-468, or BT549 cells (2 × 10^4^/well) were left to adhere for 24 h to xCELLigence E-plates. Cell proliferation was measured as a change in relative impedance, termed cell index (CI). After 24 h, the effector cells were added at the ratio of the effector to target cell (*E*:*T* = 2:1, 1:1, and 1:2) in a total volume of 200 μl. The cytotoxicity was then monitored by measuring changes in impedance as CI values recorded by the xCELLigence RTCA SP device.

### Xenograft model of TNBC

NOD/SCID/γ-chain-/- (NSG) mice were bred, treated, and maintained under pathogen-free conditions in-house under the University of Pennsylvania IACUC-approved protocols. All animals were obtained from the Stem Cell and Xenograft Core (SCXC) of the Abramson Cancer Center, University of Pennsylvania. To establish a TNBC model, 6~10-week-old female NSG mice were inoculated subcutaneously (s.c.) on the flank with 3 × 10^6^ MDA-MB-231 fluc(+) cells on day 0. After the tumors become palpable at about 3 weeks, primary human T cells were activated and transduced. After the primary human T cells were expanded for 2 weeks and the mouse tumor burden was about 200~300 mm^3^, the mice were treated with the T cells. To investigate the roles of CD27 and 4-1BB costimulated NKG2D CAR in vivo, NKG2D CAR T cells were adjusted from 90 to ~ 30% by adding UNT T cells. The route, dose, and timing of T cell injections are indicated in the individual figure legends. Tumor dimensions were measured with the calipers, and tumor volumes calculated using the formula *V* = 1/2(length × width^2^), where length is the greatest longitudinal diameter and width is the greatest transverse diameter. Animals were imaged prior to T cell transfer and 3 weeks thereafter to evaluate tumor growth. Photon emission from fluc+ cells was quantified using the “Living Image” software (Xenogen) for all in vivo experiments. Approximately 50 days after the first T cell injection, the mice were euthanized and the tumors were resected immediately.

### Bioluminescence imaging

Tumor growth was also monitored using bioluminescent imaging (BLI). BLI was conducted using Xenogen IVIS imaging system. The photons emitted from fLuc-expressing cells within the animal body were quantified using Living Image software (Xenogen). Briefly, mice bearing MDA-MB-231fLuc tumors were injected intraperitoneally (i.p.) with d-luciferin (150 mg/kg stock, 100 μL of d-luciferin per 10 g of mouse body weight) suspended in PBS and imaged under isoflurane anesthesia after 5~10 min. A pseudocolor image representing light intensity (blue, least intense; red, most intense) was generated using Living Image. BLI findings were confirmed at necropsy.

### Statistical analysis

The data are reported as means and standard deviations (SD). Statistical analysis was performed using two-way repeated-measures analysis of variance (ANOVA) for the tumor burden (tumor volume, photon counts). The Student *t* test was used to evaluate differences in absolute numbers of transferred T cells, cytokine secretion, and specific cytolysis. GraphPad Prism 5.0 (GraphPad Software) was used for the statistical calculations, where a *P* value of *P* < 0.05 was considered significant.

## Results

### Expression of NKG2D ligands on TNBC cell lines

**Fig. 1:**
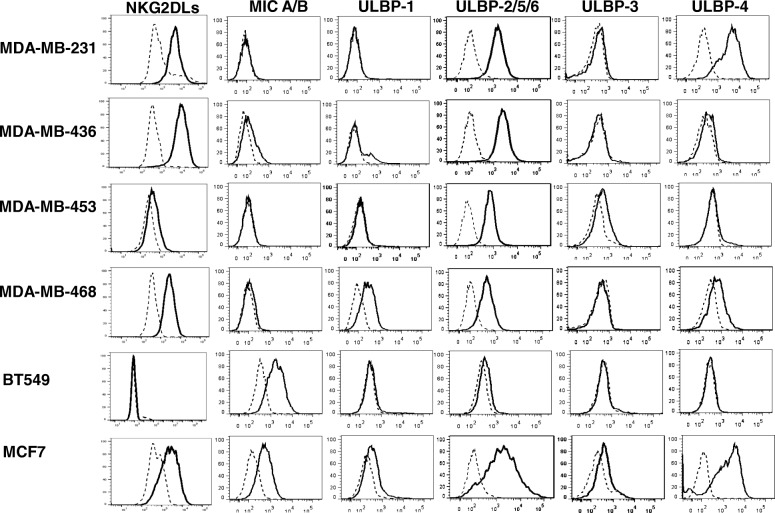
Surface expression of NKG2D ligands on TNBC cell lines. A panel of human TNBC cell lines were stained with recombinant NKG2D-Fc or specific antibodies that recognize MICA/B; ULBP-1, ULBP-2/5/6, ULBP-3, or ULBP-4 (solid line histogram); matched isotype or irrelevant recombinant protein-Fc controls (dashed line histogram) and analyzed by flow cytometry. A breast cancer cell line, MCF7, was used as a positive control

To investigate NKG2DL expression in human TNBC, we screened five TNBC cell lines for the NKG2DL expression via flow cytometry using a recombinant NKG2D receptor–human IgG1-Fc fusion protein that recognizes all ligands for this receptor. The majority of lines bound the NKG2D-Fc protein and expressed a high to moderate level of NKG2DLs, except for BT549; a lower level of NKG2D-Fc protein binding was detected on MDA-MB-453 cells (Fig. 1). AE17, a mouse malignant mesothelioma cell line [8], served as a negative control and did not express detectable human NKG2DLs (Additional file 1: Figure S1a). We next examined the expression distribution of the individual NKG2DL family members in TNBC cell lines by flow cytometry, using antibodies specific for MICA/B, or ULBP-1, ULBP-2/5/6, ULBP-3, or ULBP-4 (Fig. 1). Although varied in level of expression, all TNBC cell lines tested expressed one or more cell surface NKG2DLs. BT549 nearly exclusively expressed surface MICA/B and at high level, while a low level of MICA/B was detected on MDA-MB-436 cells. All other TNBC lines were MICA/B negative. The expression of the ULBPs on TNBC cell lines varied: ULBP1 was only expressed on MDA-MB-468 at low levels, while ULBP-2/5/6 were more often strongly expressed in all TNBC cell lines except for BT549; ULBP-3 and 4 were only found on the MDA-453 and MDA-MB-231, respectively. The breast cancer cell line MCF-7 that expressed various NKG2DLs which was previously characterized [14, 15] was used as a positive control. Together, NKG2DLs appear to be broadly expressed by TNBC cell lines, although some cell lines display a relatively low level of ligand expression.

### NKG2D CAR design and surface expression on T cells

**Fig. 2:**
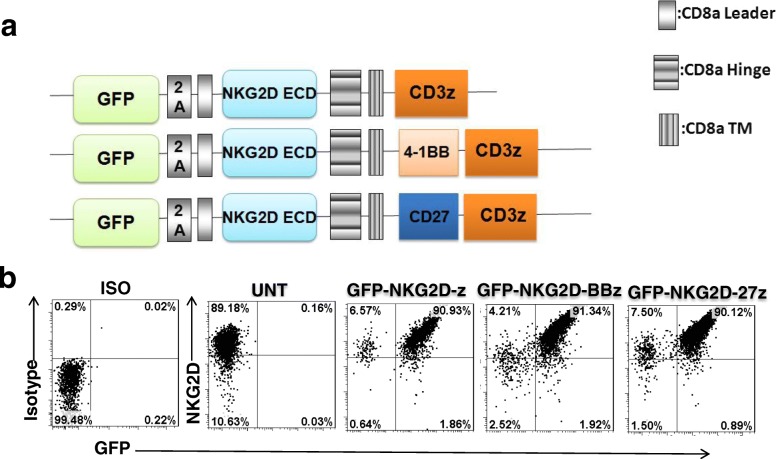
Construction and expression of NKG2D CARs. **a** Schematic illustration of the lentiviral construct for the NKG2D CARs. NKG2D CAR sequences were preceded in frame by a green fluorescent protein (GFP) sequence followed by the 2A ribosomal skipping sequence. NKG2D CAR contains the extracellular portion of the human NKG2D receptor, which linked to a CD8a hinge and transmembrane region, followed by a CD3z signaling moiety alone (GFP-NKG2D-z) or in tandem with the 4-1BB (GFP-NKG2D-BBz) or CD27 (GFP-NKG2D-27z) intracellular signaling motif. **b** NKG2D CAR and GFP coexpressed by CD3+ T cells 14 days after transduction. UNT T cells only express endogenous NKG2D

The build upon the earlier clinical efficacy of NKG2D CAR therapy in acute myeloid leukemia [7], NKG2D CARs were developed consisting of the extracellular portion of the human NKG2D receptor linked to a CD8a hinge and transmembrane region, followed by a CD3z signaling moiety alone (GFP-NKG2D-z) or in tandem with the 4-1BB (GFP-NKG2D-BBz) or CD27 (GFP-NKG2D-27z) intracellular signaling motif (Fig. 2a). Bicistronic expression vectors incorporating a 2A peptide sequence permitted dual expression analysis of GFP and the NKG2D CAR (Fig. 2b). We previously showed NKG2DLs expressed on activated T cells enrich for 4-1BB costimulated NKG2D CAR T cells [8]. Similarly, cultures of GFP-NKG2D-z, GFP-NKG2D-z-27z, and GFP-NKG2D-z-BBz CAR T cells were highly enriched for CAR+ GFP+ T cells (~ 90%) at the end of 2-week expansion in the presence of IL-2 (50 IU/ml) (Fig. 2b). Based on these results, we used GFP as a surrogate marker for detection of engineered NKG2D CAR T cells in the following experiments.

### NKG2D CAR T cells effectively recognize and eliminate TNBC cell lines in vitro

**Fig. 3:**
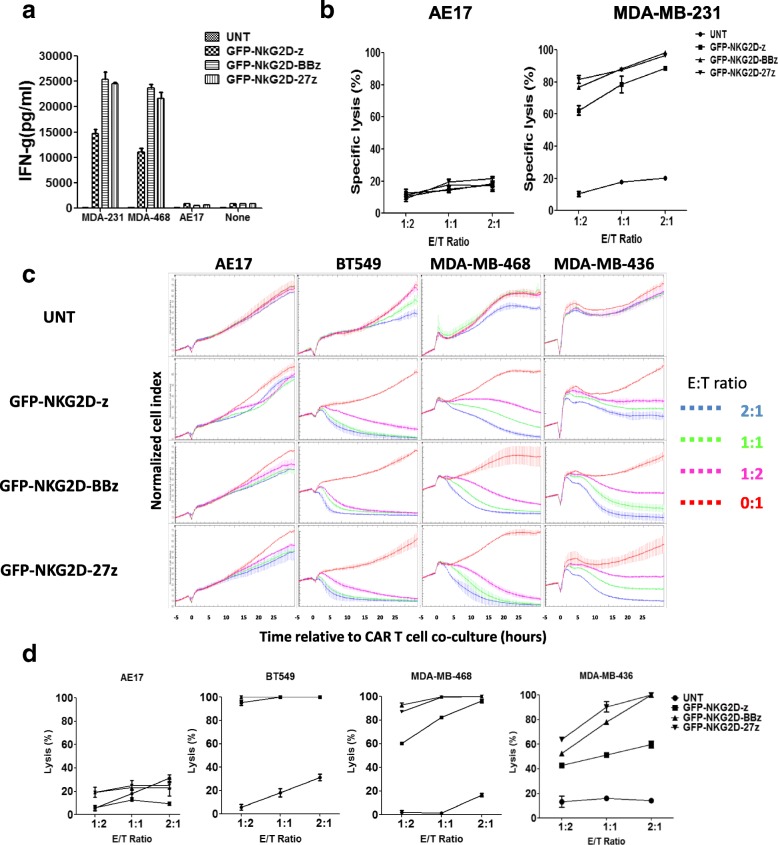
Recognition of human TNBC cells by NKG2D CAR T cells in vitro**. a** NKG2D CAR-modified T cells secrete IFN-γ during overnight co-culture with NKG2DL-expressing TNBC cells, but not NKG2DL-negative AE17 mesothelioma cells. Mean IFN-γ concentration ± SD (pg/ml) from triplicate cultures is shown. **b** Lysis of NKG2DL-expressing TNBC cells (MDA-MB-231 fluc) by NKG2D CAR T cells in an 18-h bioluminescence assay at the indicated effector-to-target (*E*/*T*) ratios. Untransduced (UNT) CD3+ human T cells and AE17 mesothelioma cells served as negative effector and target cell controls, respectively. **c** Normalized cell index(CI) plot for target cells (AE17, BT549, MDA-MB-436, and MDA-MB-468) incubated with UNT or NKG2D CAR T cells at different *E*:*T* ratios for 24 h. When seeded alone, target cells adhere to the plate and proliferate, increasing the CI readout (red lines). When T cells added to target cells, NKG2CD CAR T cells cause cell cytolysis and subsequent progressive decrease in CI. *Y*-axis is the normalized CI generated by the RTCA software and displayed in real time. *X*-axis is the time of cell culture and treatment time in hour. Mean values of the CI were plotted ± standard deviation. **d** The cell index plot is converted to a % Lysis plot by the xCELLigence Immunotherapy Software

To evaluate the anti-tumor function of NKG2D CAR-T cells in vitro, primary human T cells and TNBC cells were co-cultured and CAR T cell reactivity measured by proinflammatory cytokine secretion. NKG2D CAR-T cells recognized TNBC cell lines MDA-MB-231and MDA-MB-468 and secreted high levels of IFN-γ, but not when stimulated with the NKG2DL (−) cell line AE17 (Fig. 3a), illustrating the requirement for antigen specificity for CAR-T cell activity. Invariably, increased quantities of secreted IFN-γ were detected when NKG2D-27z and NKG2D-BBz CAR-T cells where stimulated with the NKG2DL (+) target cells, relative to the first-generation NKG2D-z CAR T cells (Fig. 3a).

We next evaluated the cytotoxicity of NKG2D CAR T cells against the NKG2DL (+) TNBC cell line, MDA-MB-231fluc, using an overnight luminescence-based assay. Compared to the control untransduced T cells, NKG2D CARs T cells demonstrated significant cytotoxicity against firefly luciferase (fLuc) expressing MDA-MB-231 cells. When the *E*:*T* ratio was as low as 1:2, the cytotoxicity was more than 60% and increased as the *E*:*T* ratio increased (Fig. 3b). The NKG2DL (−) cell line AE17 fLuc was not lysed by NKG2D CAR T cells. Similar to cytokine production results, costimulated NKG2D-BBz or NKG2D-27z CAR-T cells demonstrated enhanced cytotoxicity compared to their first-generation counterparts (Fig. 3b).

Similarly, xCELLigence cytotoxic data showed that 4-1BB or CD27 costimulated NKG2D CAR-T cells were cytotoxic toward NKG2DL (+) MDA-MB-468, MDA-MB-436 cells in a time- and *E*:*T* ratio-dependent manner, while untransduced T cells did not inhibit the growth of these cells (Fig. 3c). As expected, NKG2D-z CAR T cells were less efficient in killing NKG2DL (+) target cells and required higher *E*/*T* ratios to achieve efficient response (Fig. 3c, d). Interestingly, after 24 h of co-culture, addition of any iteration of NKG2D CART cells caused BT549 cells to detach from the culture plate, consequently reducing cell index value, suggested BT459 cells were lysed efficiently even at low 1:1 *E*/*T* ratio (Fig. 3c, d), although these cells only express MIC A/B but no detectable expression of NKG2DLs measured by NKG2D-Fc (Fig. 1). This further suggests that BT549 cells may be more sensitive than MDA-MB-436 and MDA-MB-438 cells to cytolysis by CAR T cells. Similar to the luciferase release-based cytotoxicity assays, the NKG2DL (−) cell line AE17 was not lysed by NKG2D CAR T cells (Fig. 3c, d).

### IL-2 promotes expansion and enrichment of NKG2D-redirected CAR T cells

**Fig. 4:**
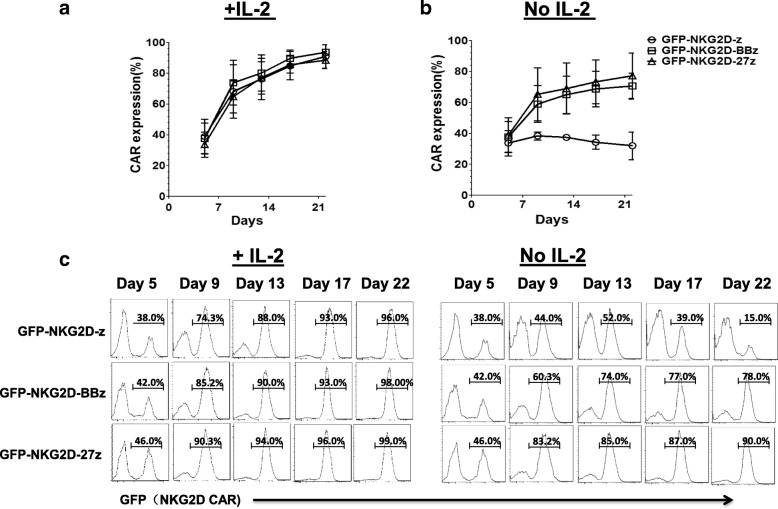
NKG2D CAR (GFP) expression on T cells in the presence and absence of IL-2. Percentages of NKG2D CAR (GFP+) cells, presented as the mean ± SD were derived from three independent donors during a 3-week culture period in the presence of IL-2 (50 IU/ml) (**a**) or absence of IL-2 (**b**). **c** The kinetic monitoring of GFP (NKG2D CAR) expression on one representative donor of three in the presence or absence of IL-2

During the culture of NKG2D CAR T cells in the presence of IL-2, we consistently observed the temporal enrichment of both the first and second generation of NKG2D CARs, with the frequencies of CAR+ T cells increasing temporally. To investigate the influence of IL-2 on this NKG2D CAR enrichment phenomenon, CAR T cells were washed free of IL-2 using PBS on day 5 after activation and transduction, and then cultured in complete medium in the presence or absence of exogenous IL-2 (50 IU/ml). CAR T cell count and expression level were monitored for additional 2 weeks (Additional file 1: Figure S1b). Consistently, in this assay from three different donors whose untransduced T cells express NKG2DLs when activated by anti-CD3/28 beads (Additional file 1: Figure S1c), GFP-expressing NKG2D-z, NKG2D-BBz, and NKG2D-27z CAR T cells expanded more than 300-fold (Additional file 1: Figure S1d) and were highly enriched for CAR+ cells during prolonged culture in the presence of IL-2 (Fig. 4a). Only ~ 30% of T cells were positive for GFP expression on day 5, but were preferentially enriched to more than 95% GFP+ after an additional 2 weeks of culture (Fig. 4a). In stark contrast, in the absence of IL-2, NKG2D CAR T cells did not expand well (Additional file 1: Figure S1d) and only T cells that expressed the costimulated GFP-NKG2D-BBz and GFP-NKG2D-27z CARs were still highly enriched for CAR+ cells. In contrast, the frequency of GFP-NKG2D-z CAR T cells remained stable at ~ 30% over this entire time, suggesting that a costimulatory signal may be required for CAR enrichment in the absence of IL-2. The kinetic monitoring of surface CAR expression on one representative donor in the presence or absence of IL-2 is shown in Fig. 4c.

### CD25 high expression on first-generation NKG2D CAR T cells is essential for CAR enrichment in the presence of IL-2

**Fig. 5:**
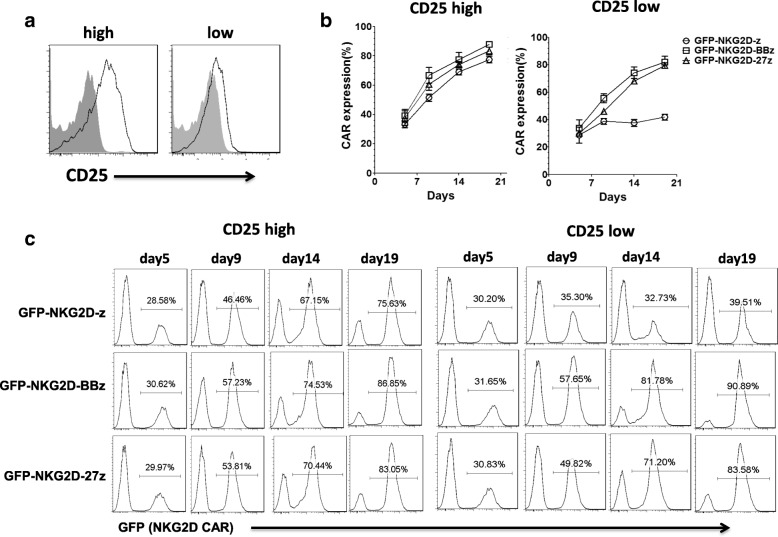
CD25 high expression on GFP-NKG2D-z CAR T cells is essential for enrichment in the presence of IL-2. **a** One representative donor NKG2D CAR T cells expressed high level of CD25 and low level of CD25 by depleting human CD25 with magnetic separation on day 5 (4 days post transduction). **b** Percentages of CD25^high^ and CD25^low^ expressing GFP+ (NKG2D CAR) cells, presented as the mean ± SD, were derived from three independent donors during a 3-week culture period in the presence of IL-2 (50 IU/ml). **c** The kinetic monitoring of CD25^high^ and CD25^low^ expressing GFP (NKG2D CAR) expression on one representative donor of three in the presence of IL-2

We previously showed that the enrichment of CAR may due to the “T cell fratricide” of the 4-BB costimulated NKG2D CAR T cells interacting with NKG2DLs expressed on activated T cells [8]. However, in the presence of IL-2, the first-generation NKG2D-z CAR was also highly enriched for CAR (+) cells during culture in vitro. This result suggested that IL-2 stimulation may promote the CAR T cell expansion and upregulate NKG2DLs to enrich NKG2D CAR T cells even without costimulation. To test our hypothesis, we sought to investigate whether CD25 (IL-2 receptor-alpha chain) expression is associated with NKG2D CAR enrichment. On day 5 after activation, 4 days post transduction, nearly all of NKG2D CAR+ T cell population expressed a high level of CD25 (Fig. 5a, *left*). All NKG2D-z, NKG2D-BBz, and NKG2D-27z CAR T cells that expressed high levels of CD25 were preferentially enriched to ~ 80% after an additional 14 days of culture (Fig. 5b, *left*).). We then separated out the CD25^low^ NKG2D-z, NKG2D-BBz, and NKG2D-27z CAR T cells by depleting human CD25+ cells by magnetic separation 4 days post transduction (Fig. 5a, *right*). NKG2D-BBz and NKG2D-27z CAR T cells that were CD25^low^ were still ~ 80% enriched after additional 14 days culture in the presence of IL-2 (Fig. 5b, *right*). Alternatively, NKG2D-z CAR T cell expression frequency in the CD25^low^ population ranged between 30 and 40%, which was substantially lower than costimulated NKG2D CAR expression, suggesting that high CD25 expression is essential for IL-2-driven NKG2D CAR enrichment in the absence of costimulatory signal domains and that the CAR costimulatory domains are sufficient to drive NKG2D CAR enrichment even when surface CD25 expression levels are low. The kinetics of CAR expression in T cells with CD25^high^ or CD25^low^ expression in the presence of IL-2 is shown for one representative donor (Fig. 5c).

CAR T cells may become exhausted due to the persistent antigen exposure or tonic signaling of the CAR [16]. To ascertain whether T cell exhaustion develops during NKG2D CAR T cell expansion, we measured the expression of the two more abundantly expressed inhibitory receptors on T cells, TIM3 and PD-1. Similar to the untransduced T cells, NKG2D CAR T cells expressed low levels of the PD1 and TIM3 (Additional file 2: Figure S2a). Although these CAR T cells were highly enriched by “T cell fratricide” in the presence of IL-2, they did not become exhausted. Interestingly, first-generation NKG2D-z CAR T cells expressed relatively higher levels of PD-1 and TIM-3 compared to costimulated NKG2D-BBz or NKG2D-CD27z CAR T cells, and even untransduced T cells. High levels of PD-1 and TIM3 were however expressed on activated T cells stimulated by anti-CD3/CD28 beads (Additional file 2: Figure S2a). We also measured 4-1BB and PD-1 expression on NKG2D CAR T cells because the presence of the target antigen on T cells may sustain high tonic signaling which leads to increased activation marker and exhaustion [16]. Similar to untransduced T cells, CAR T cells lacked 4-1BB expression (Additional file 2: Figure S2b) indicating a lack of tonic signaling and suggesting insufficient stimulation by NKG2DLs, possibly due to the transient nature of expression of NKG2DLs in activated T cells. Alternatively, the small subsets of 4-1BB expressing CAR T cells might preferentially express NKG2DLs and accordingly be killed by other NKG2D CAR T cells leading to a “CAR T cells-fratricide and auto-stimulation/enrichment” cycle. Incubation of NKG2D CAR T cells with the MDA-MB-231 cell line resulted in robust activation-induced upregulation of 4-1BB restricted to CAR+ T cells and a significant upregulation of PD-1 on CAR+ cells after 24 h of incubation (Additional file 2: Figure S2b). Similar to the above experiments, first-generation NKG2D-z CAR T cells expressed relatively higher levels of PD-1 compared to costimulated NKG2D-BBz or NKG2D-CD27z CAR T cells during culture or when stimulated with MDA-MB-231 cells (Additional file 2: Figure S2b). Together, these data suggested that NKG2D CAR T cells do not become exhausted during ex vivo expansion and that 4-1BB or CD27 costimulation may enable T cells to resist immune exhaustion, consistent with a prior report focused on 4-1BB [16].

### Control of TNBC by costimulated NKG2D CAR T cells in vivo

**Fig. 6:**
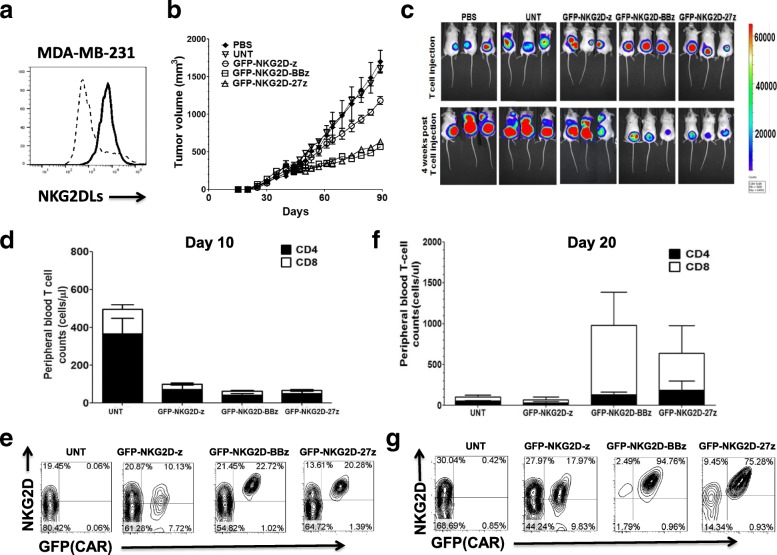
Costimulated NKG2D CAR T cells inhibit tumor growth of TNBC in vivo*.* **a** NKG2D ligand expression on TNBC cell line MDA-MB-231 detected by NKG2D-Fc chimeric protein. **b** Growth curve of MDA-MB-231 tumor (*n* = 5) treated with the control PBS, UNT T cells (3 × 10^7^), or NKG2D CAR T cells (3 × 10^7^, ~ 30% CAR+) by intravenous injection 40 and 45 days post tumor inoculation. At the end of the experiment, the tumors treated with GFP-NKG2D-BBz or GFP-NKG2D-27z CAR T cells were significantly smaller than those in the control group and GFP-NKG2D-z group (*P* < 0.001). **c** Bioluminescence images was applied to monitor and quantify MDA-MB-231 fLuc(+) tumor growth in NSG mice immediately before and 3 weeks after first CAR-T cell injection. **d** CD4+ and CD8+ GFP-NKG2D-BBz and GFP-NKG2D-27z CAR T cells were initially present at low numbers in peripheral circulation, suggesting NKG2DL-specific CAR T cell migration to specific tumor locales. Mean cell concentration (cells/ul) ± SEM for all evaluable mice in each treatment group is shown (*n* = 5). **e** NKG2D-z CAR, but not costimulated NKG2D CAR, was poorly expressed on the surface of transduced (GFP+) T cells, suggesting CAR downregulation. **f**, **g** CD27 and 4-1BB signaling enhances the survival of circulating human CD4+ and CD8 +T cells in vivo 3 weeks after first dose of T cell infusion(*P* < 0.01) and costimulated NKG2D CAR expression on the T cell surface is stable and increased in vivo

We next evaluated the therapeutic efficacy of these NKG2D CAR T cells against human TNBC in vivo*.* We first generated a xenograft model of TNBC by injecting 3 × 10^6^ luciferase-labeled NKG2DL-expressing MDA-MB-231 cells (Fig. 6a) subcutaneously in NSG mice. When tumors reached a mean volume of ~ 300 mm^3^, mice were assigned to the following treatment groups: administration of PBS, untransduced T cells, or NKG2D CAR (+) T cells on day 40 and day 45 after tumor implantation by tail-vein injection. MDA-MB-231 tumors in the control mice group treated with PBS or untransduced T cells grew progressively beyond the time of T cell transfer as measured by caliper-based sizing and bioluminescent imaging (BLI) (Fig. 6b, c). These mice had to be euthanized due to high tumor burden by day 90. Tumor growth was modestly delayed in mice receiving GFP-NKG2D-z T cells, compared with control groups at the latest evaluated time point. In contrast, mice receiving GFP-NKG2D-BBz or GFP-NKG2D-27z CAR T cells were protected from rapid progression (Fig. 6b, c), which was significantly better than NKG2D-z T cells (*P* < 0.001).

Peripheral blood was collected from tumor-bearing mice and quantified for persistence of infused human T cells. Ten days after the first injection of T cells, human CD4+ T cell counts were higher compared to CD8+ T cell counts in the circulation and both CD4+ and CD8+ T cells in GFP-NKG2D-z, GFP-NKG2D-BBz, and GFP-NKG2D-27z CAR cohorts were present in lower numbers in comparison to untransduced T cells, suggesting early NKG2DL-specific CAR T cell migration to specific tumor sites (Fig. 6d). NKG2D CAR expressing T cells were detectable in the peripheral blood. The level of CAR expression by NKG2D-BBz and NKG2D-27z T cells was high compared to the expression detected on NKG2D-z CAR T cells, which poorly expressed on the surface of transduced (GFP+) T cells, suggesting CAR downregulation (Fig. 6e). Twenty days after first T cell injection, significant CD4+ and CD8+ T cell expansion was now detected in the peripheral blood of mice receiving GFP-NKG2D-BBz or GFP-NKG2D-27z CAR T cells compared to untransduced and GFP-NKG2D-z treatment groups, indicating roles for 4-1BB and CD27 in T cell survival in vivo (Fig. 6f). For the costimulated CAR groups tested, the CD8 T cell counts were higher than the CD4 T cell counts. Analysis of the CD8 data in these two groups revealed statistically similar cell counts in the GFP-NKG2D-BBz CAR T cell-treated group compared to GFP-NKG2D-27z group (*P* > 0.05) (Fig. 6f). These results indicate that both 4-1BB and CD27 costimulation augment CD8 T cell persistence but also suggest 4-1BB may be slightly superior for CD8+ T cells. Similar to what we observed in vitro, 4-1BB or CD27 costimulated NKG2D CAR T cells maintained a high level of surface CAR expression in vivo without the need for additional exogenous IL-2 support; this was not the case for the NKG2D-z CAR (Fig. 6g). These data suggest that costimulated NKG2D CAR expression on the T cell surface is stable and increased in vivo, even after antigen recognition and the proliferation of the CAR T cells*.* Together, these results support the use of costimulated NKG2D-BBz or NKG2D-27z CAR T cells as a cellular modality for enhanced treatment of TNBC in vivo.

## Discussion

The expression of NKG2DLs on many primary tumor cells and immunosuppressive cells (e.g., T regulatory cells and myeloid-derived suppressor cells) within the tumor microenvironment makes them attractive targets for the development of novel therapeutics [17]. Targeting NKG2DLs with NKG2D CAR T cells has been shown to induce tumor elimination and long-term tumor-free survival in various tumor models [18–20]. NKG2DLs are frequently expressed in breast cancer [21], and we found positive surface expression of NKG2DLs on all (5/5) human TNBC cell lines tested by flow cytometry. Based on these results, we rationalized the extension of our NKG2D CAR T cell-based immunotherapy approach to TNBCs.

NKG2D CAR T cells secreted IFN-γ upon stimulation with NKG2DL (+) tumor cells and displayed potent cytolytic capacity in vitro against NKG2DLs+ TNBC cells, even at low *E*/*T* ratios. Consistently, the production of proinflammatory cytokines and cytolytic capacity by NKG2D-BBz and NKG2D-27z CAR-T cells was substantially increased after co-culture with NKG2DL (+) TNBC cell lines, compared with NKG2D-z T cells. Mechanisms accounting for increased effector function by 4-1BB and CD27 costimulated CAR-T cells in vitro appear linked in part to their ability to resist antigen-induced cell death (AICD) as we reported previously [11]. These in vitro tumor killing findings further support the notion that NKG2DLs have promise as novel immunotherapy targets for TNBCs, which currently lack effective targeted therapies. Indeed, two injections of 4-1BB or CD27 costimulated NKG2D CAR T cells exhibited in vivo anti-tumor effects in a highly invasive MDA-MB-231 xenograft model of human TNBC, compared to the first-generation NKG2D CAR T cells. Consistent with clinical observations [22], tumor regression was associated with enhanced T cell persistence in vivo. The greatest number of CAR T cells persisting in the blood 20 days after the first T cell dose was observed in those animals administered NKG2D-BBz and NKG2D-27z CAR-T cells followed by NKG2D-z and untransduced T cells, indicating that simultaneous TCR CD3 signaling and 4-1BB or CD27 costimulation triggered by CAR ligation with antigen improves upon TCR signaling alone, implicating a role for 4-1BB and CD27 costimulation in memory T cell formation in vivo. Results of comparative in vivo studies of CARs containing these various costimulatory domains demonstrated that both 4-1BB and CD27, members of the TNFR superfamily, enhances T cell anti-tumor activity and persistence.

In this study, costimulated NKG2D CAR T cells failed to completely eradicate large, established tumors. Major studies suggest that administration of exogenous IL-2 may be necessary for the anti-tumor activity for the first-generation CAR T cells that lack costimulatory domains [23, 24]. We have previously found that the administration of IL-2 has little to no anti-tumor effect on human CAR T cells activity in immunodeficient mice, if the CAR contains a costimulatory domain [25]. Therefore, the omission of exogenous IL-2 is not likely the primary factor accounting for the suboptimal anti-tumor response in this study. The efficacy of NKG2D CAR T cell treatment for TNBC may also be influenced by surface antigen expression level as suggested previously [26] in the same MDA-MB-231 tumor model. Although MDA-231 cells express various NKG2DLs, a previous study [27] suggested that NKG2D can bind to only one ligand at a time. In addition, NKG2D displays varying affinities for its ligands that ranges between 600 nM and 1.1 mM [28] and NKG2D CAR may preferentially bind to the higher affinity form of ligands. Therefore, further investigation of affinity of each ligand’s binding to NKG2D receptor and selecting patients with higher NKG2DL expression may help to enhance the anti-tumor activity of NKG2D CAR T cells in clinical studies. Other antigens such as folate receptor-a [26], mesothelin [29], and TEM8 [30] were also investigated as CAR targets for TNBCs with mixed efficacy results reported. Targeting solid tumors, like TNBC, is still challenging, as it may be greatly hampered clinically by the immunosuppressive microenvironment and the inefficient homing of CAR T cells to tumor sites [31]. Combining CAR T cells with other therapies like CTLA-4 and PD-1 inhibitor offers the potential to improve anti-tumor effects.

One intriguing finding from this study is that in the presence of exogenous IL-2, a potent cytokine stimulator of activated effector lymphocyte expansion and proliferation, NKG2D CAR T cells, undergoes substantial long-term expansion without evidence of prolonged detriment from fratricide in vitro. In addition, the NKG2D CAR (+) T cell population was enriched during prolonged culture, suggesting a possible auto-stimulatory effect through endogenous expression of induced NKG2DLs after T cell stimulation. These findings are similar to a CD28 costimulated CD5-specific CAR [32], which kills both CD5(+) autologous T cells and malignant T cell lines, yet results in limited and transient T cell fratricide. However, by incorporating 4-1BB, CD27, OX40, CD30, CD28, ICOS, or HVEM into the CD5 CAR, a more recent study suggested that only CD5 CARs costimulated with non-TNFR domains (CD28, ICOS, or non-costimulated) overcome impaired the expansion of CAR T cells and resulted in complete or partial downregulate CAR expression [33]. Unlike the CD5 CAR platform, which differs significantly in expansion and transduction methodology and antigen specificity, our NKG2D CAR T cells undergo dramatic expansion and preferentially enrich for CAR expressing T cells during culture. Here, exogenous IL-2 promoted the expansion and enrichment of NKG2D-z, NKG2D-BBz, and NKG2D-27z CAR (+) T cells, which was dependent on a high expression level of the IL-2 receptor-alpha subunit, CD25. Without IL-2 support, T cells expressing any of the NKG2D constructs did not expand well and only NKG2D CAR (+) T cells containing 4-1BB or CD27 costimulatory domains enriched for CAR-positivity, demonstrating a benefit from 4-1BB and CD27 costimulation in vitro. These results suggest that incorporation of costimulatory domains into CAR constructs promotes preferential T cell survival and resistance to AICD by upregulating anti-apoptotic proteins as suggested previously [11]. These data also support the three-signal hypothesis that optimal T cell activation and expansion involves T cell receptor (TCR) activation (signal 1) in addition to costimulatory receptor engagement (signal 2) and cytokine receptor engagement (signal 3) [34]. Although NKG2DLs can be expressed on active T lymphocytes, our NKG2D CARs do not elicit tonic signaling, as seen with other CARs reported previously [16, 35]. In one report [35], tonic signaling CAR T cells displayed sustained proliferation for up to 3 months, resulting from autocrine CAR crosslinking that is independent of cognate antigen and did not require the addition of exogenous cytokines or feeder cells after a single stimulation of the TCR and CD28. NKG2D-z, NKG2D-BBz, and NKG2D-27z CAR (+) T cells do not undergo prolonged and unfettered proliferation. Whether regulated proliferation in NKG2D CAR T cells is impacted by the selection of the 4-1BB and CD27 TNFR costimulatory domains, and not CD28, is not known. However, in the setting of tonic CAR signaling, 4-1BB costimulation reduces exhaustion induced by persistent CAR signaling and augments anti-tumor activity in vivo, while CD28 costimulation does not [16].

In light of the therapeutic potential of NKG2D CAR T cells for TNBC and because of the potential for expression of NKG2DLs on healthy tissues, the concerns about potential “on-target, off-tumor” toxicity must be considered. VanSeggelen et al. [36] described lethal toxicity in mice treated with murine NKG2D CAR T cells. This toxicity was both CAR construct and strain dependent and was exacerbated upon the use of a lymphodepleting conditioning regimen. These results suggested that predicting the toxicity of NKG2D CAR approach becomes especially problematic because of the diversity of target ligands. Moreover, both efficacy and toxicity will likely be affected by the ligand tissue distribution, density, and affinity with which NKG2D binds the respective ligand. None of these parameters are very well characterized and likely vary greatly between mice and humans [37]. However, results from a phase I clinical study [38] testing the safety of NKG2D CAR-T cells in patients with AML/MDS and multiple myeloma (ClinicalTrials.gov Identifier: NCT03018405) are promising with no reported cases of cytokine-release syndrome, CAR T cell-related neurotoxicity, autoimmunity, or patient death. It is important to have adequate safety data in multiple clinical studies using different NKG2D CAR constructs. In the current ongoing clinical trial [38], the full-length NKG2D is used in a CAR design that incorporates Dap10 signaling, which was showed the enhanced CAR expression and toxicity [36]. Notably, one recent case study reports that NKG2D-based chimeric antigen receptor therapy safely induced remission in a patient with relapsed/refractory acute myeloid leukemia [7]. Our NKG2D CAR construct contains 4-1BB and lacks DAP10, yet still possesses potent anti-tumor capacity against TNBC. Therefore, future clinical studies are warranted.

## Conclusions

In conclusion, we have identified a self-enrichment phenomenon of NKG2D CAR in vitro*,* which does not affect T cell expansion. These NKG2D CAR T cells can effectively target NKG2DLs expressing TNBC cells in vitro, and CD27 or 4-1BB costimulated CAR T cells can significantly reduce tumor growth in vivo. NKG2D CAR T cell therapy may provide novel treatment options for patients with TNBCs and may be amenable to combination with immune checkpoint blockade, cytokines, and other strategies to transform this approach from being “promising” to being “effective” treatments for TNBC and other solid tumors.

## Additional files

Additional file 1: **Figure S1.** a Schematic of the monitoring NKG2D CAR expression procedure in the presence or absence of IL-2. NKG2DLs are expressed on activated CD8+ and CD8-(CD4)T cells after 4 days activation by anti-CD3/28 beads. b Results for three independent donors are shown and irrelevant folate receptor-alpha (FRA)-Fc protein was used as negative control. c T cell expansion folds in the presence of IL-2 (50 IU/ml) or absence of IL-2. (PPTX 226 kb)

Additional file 2: **Figure S2.** a Expression of PD-1 and TIM-3 markers of exhaustion in T cells during culture.Anti-CD3/28 beads activated NKG2D-27z CAR T cells (15 h stimulation) were used as positive control for expression of PD-1 and TIM-3. b Expression of CD137 and PD-1 in T cells during culture and when co-cultured with MDA-MD-231 cells. (PPTX 147 kb)

## Abbreviations

7-AAD: 7-Aminoactinomycin D
AICD: Antigen-induced cell death
CAR: Chimeric antigen receptor
ECD: Extracellular domain
ER: Estrogen receptor
GFP: Green fluorescent protein
HER2: Human epidermal growth factor receptor 2
i.p.: Intraperitoneally
IL-2R: IL-2 receptor
MIC: MHC class I-related chain
NKG2D: Natural-killer group 2, member D
NKG2DLs: NKG2D ligands
NSG: NOD/SCID/γ-chain-/-
PR: Progesterone receptor
s.c.: Subcutaneously
TCR: T cell receptor
TNBC: Triple-negative breast cancer
ULBP/RAET: UL16-binding protein, or retinoic acid early transcript
