# Supplementary figures and images for: Control of triple-negative breast cancer using ex vivo self-enriched, costimulated NKG2D CAR T cells
Source: J Hematol Oncol. 2018 Jul 6;11:92. doi: 10.1186/s13045-018-0635-z (PMC6035420; doi:10.1186/s13045-018-0635-z)

## Slide 1
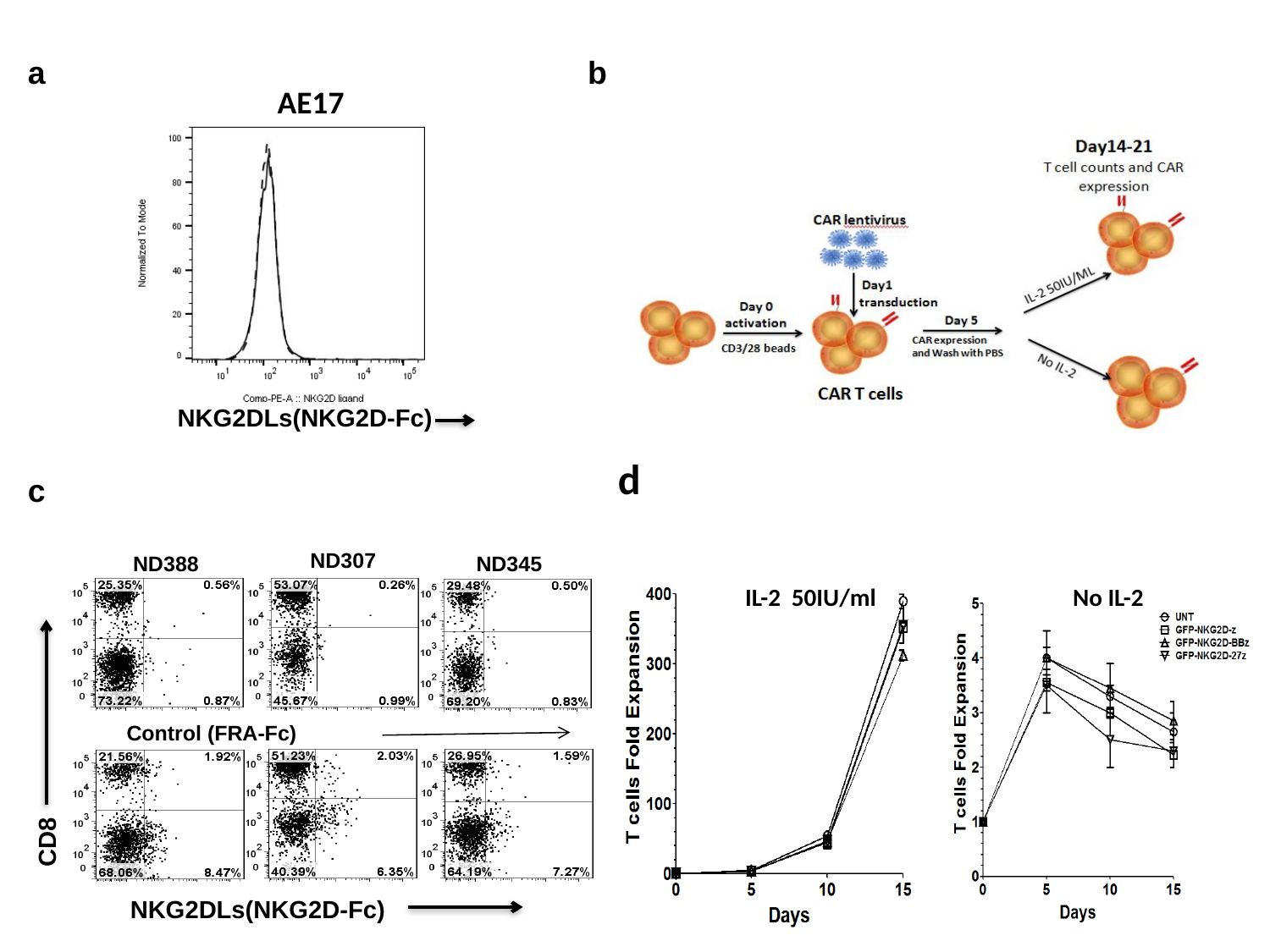

a
b
AE17
NKG2DLs(NKG2D-Fc)
d
c
ND307
ND388
ND345
Control (FRA-Fc)
CD8
NKG2DLs(NKG2D-Fc)
IL-2 50IU/ml
No IL-2

Supplement: Supplementary file 1 — Figure S1. a Schematic of the monitoring NKG2D CAR expression procedure in the presence or absence of IL-2. NKG2DLs are expressed on activated CD8+ and CD8-(CD4)T cells after 4 days activation by anti-CD3/28 beads. b Results for three independent donors are shown and irrelevant folate receptor-alpha (FRA)-Fc protein was used as negative control. c T cell expansion folds in the presence of IL-2 (50 IU/ml) or absence of IL-2. (PPTX 226 kb) [file 13045_2018_635_MOESM1_ESM.pptx]
