# Supplementary material for: Control of triple-negative breast cancer using ex vivo self-enriched, costimulated NKG2D CAR T cells
Source: J Hematol Oncol. 2018 Jul 6;11:92. doi: 10.1186/s13045-018-0635-z (PMC6035420; doi:10.1186/s13045-018-0635-z)
Supplement: Supplementary file 2 — Figure S2. a Expression of PD-1 and TIM-3 markers of exhaustion in T cells during culture.Anti-CD3/28 beads activated NKG2D-27z CAR T cells (15 h stimulation) were used as positive control for expression of PD-1 and TIM-3. b Expression of CD137 and PD-1 in T cells during culture and when co-cultured with MDA-MD-231 cells. (PPTX 147 kb) [file 13045_2018_635_MOESM2_ESM.pptx]

## Slide 1
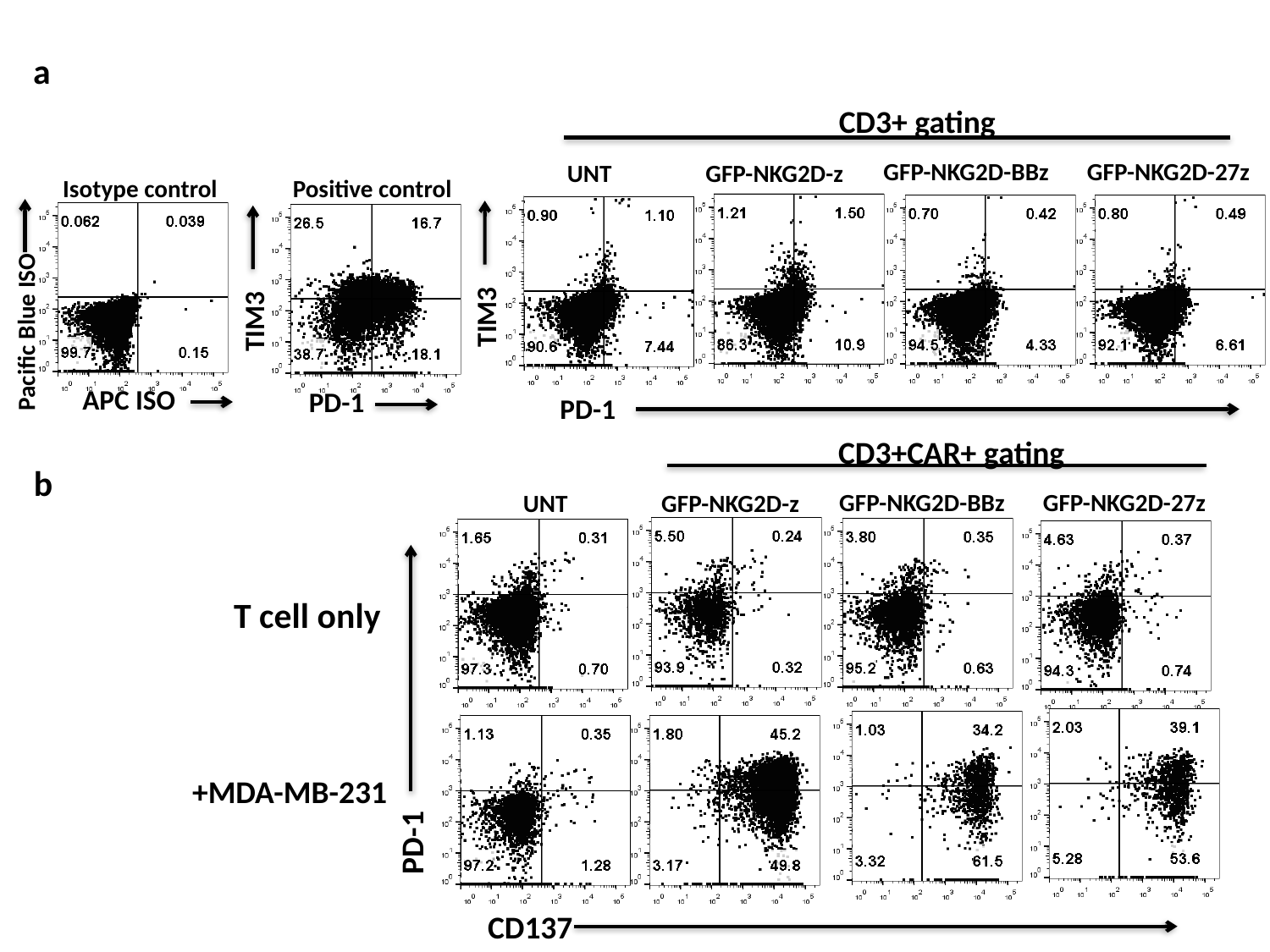

a
CD3+ gating
GFP-NKG2D-BBz
GFP-NKG2D-27z
UNT
GFP-NKG2D-z
Isotype control
Positive control
Pacific Blue ISO
TIM3
TIM3
APC ISO
PD-1
PD-1
CD3+CAR+ gating
GFP-NKG2D-BBz
GFP-NKG2D-27z
UNT
GFP-NKG2D-z
PD-1
CD137
T cell only
+MDA-MB-231
b
